# Association of triglyceride-glucose index trajectories with the risk of worsening heart failure in elderly patients with chronic heart failure and type 2 diabetes: a competing risk analysis

**DOI:** 10.1186/s12933-025-02687-8

**Published:** 2025-03-21

**Authors:** Yingying Lai, Cailong Lin, Xindong Liu, Yuting Liu, Hua Cai, Nannan Zhao, Yushuo Gao, Ziyi Yi, Jianyu Huang, Min Li, Lin Xu

**Affiliations:** 1 School of Public Health, Guangdong Pharmaceutical University, Guangzhou, China; 2 Department of Geriatric Cardiology, General Hospital of Southern Theater Command, Guangzhou, China; 3 Guangdong Pharmaceutical University, Guangzhou, China; 4 The First School of Clinical Medicine, Southern Medical University, Guangzhou, China

**Keywords:** Trajectory, Over 60 years old, Triglyceride-glucose index, Worsening heart failure, Chronic heart failure, Type 2 diabetes

## Abstract

**Background:**

The triglyceride-glucose index serves as a dependable biomarker for gauging insulin resistance linked to cardiovascular disease. Our study was designed to investigate how the trajectory of the triglyceride-glucose index relates to the risk of worsening heart failure and overall mortality in patients aged 60 years and older with chronic heart failure and type 2 diabetes.

**Methods:**

This study enrolled 466 patients who had ≥ 3 medical exams. The formula for calculating the triglyceride-glucose index was ln (fasting triglycerides [mg/dL] × fasting blood glucose [mg/dL]/2). The trajectory of the triglyceride-glucose index in longitudinal analysis was analyzed via linear mixed models. The relationships between the trajectory of the TyG index and the risk of worsening heart failure and overall mortality were analyzed via competing Cox regression analysis and mixed-effects Cox regression analysis.

**Results:**

After the variables adjustment, compared with the first quartile group, the adjusted hazard ratios for worsening heart failure in top quartile group were 2.40 (1.35–3.28) for 10-year follow-up, and 2.09 (1.22-3.58) for overall follow-up duration. The adjusted hazard ratios for overall mortality in top quartile group were 1.99 (1.56–3.14) for 10-year follow-up, and 1.87 (1.22–2.88) for overall follow-up duration. Compared with the low decreasing trajectory, adjusted hazard ratios for worsening heart failure of high decreasing trajectory were 1.37 (1.10–1.71) for the 5-year follow-up, 1.78 (1.10–2.88) for 10-year follow-up, and 1.67 (1.04–2.68) for overall follow-up duration. The adjusted hazard ratios for overall mortality were 2.16 (1.39–3.35) for 10-year follow-up, and 2.23 (1.46–3.40) for overall follow-up duration.

**Conclusion:**

During follow-up, a higher baseline level of TyG index and a high decreasing trajectory were independently associated with long-term worsening heart failure and an increased risk of overall mortality.

**Supplementary Information:**

The online version contains supplementary material available at 10.1186/s12933-025-02687-8.

## Background

As the population ages more rapidly, the burden of cardiovascular diseases (CVDs) has become a significant concern for both society and families [1]. Heart failure (HF), which is particularly prevalent among elderly individuals, represents the final stage of the progression of heart disease [2]. There are 8.9 million people living with HF in China, and the average hospital cost for these patients is one of the highest among patients with CVD [3]. There are three main reasons for the management challenges in these patients. First, while significant advances have been made in medical and surgical treatments, the 5-year mortality rate in patients with HF is still greater than 50% [4–6]. Second, the aging population has exacerbated the incidence and prevalence of HF. The average age of HF patients in China is 71.0 ± 12.7 years [3]. Finally, comorbidities such as hypertension (58.2%), diabetes (DM) (28.1%), and chronic kidney disease (CKD) (12.6%) increase the burden of HF [7]. HF and type 2 diabetes (T2DM) are risk factors for each other [8]. Owing to long-standing abnormalities in blood glucose and insulin metabolism, patients with HF and T2DM are more likely to face worsening prognoses and increased hospital costs [9]. Identifying high-risk factors in elderly HF patients with T2DM is crucial for timely intervention and alleviating the disease burden.

High levels of insulin insensitivity (II) remain linked to a heightened likelihood of HF even after accounting for conventional risk factors [10]. The TyG index has proven to be a dependable indicator for evaluating insulin resistance (IR) and acts as a notable prognosticator of diverse detrimental cardiovascular consequences [11–13]. Notably, it is strongly correlated with the prognosis of multiple CVDs, including hypertension, coronary heart disease (CHD), arteriosclerosis, HF, and stroke [12, 14–19]. Nonetheless, few studies have explored the prolonged alterations in the TyG index and its correlation with the concurrent presence of HF and T2DM, especially with respect to the peril of unfavorable clinical results in geriatric patients [20–22].

Therefore, we conducted this retrospective cohort study. Our aim was to gain an understanding of the relationships between the baseline TyG index and its trajectory and the risk of worsening heart failure (WHF) and overall mortality in senior patients with chronic heart failure (CHF) and T2DM during various follow-up periods. The results of this study are expected to provide unique insights into clinical practice and health policy development in elderly patients with CHF and T2DM.

## Method

### Study population

**Fig. 1:**
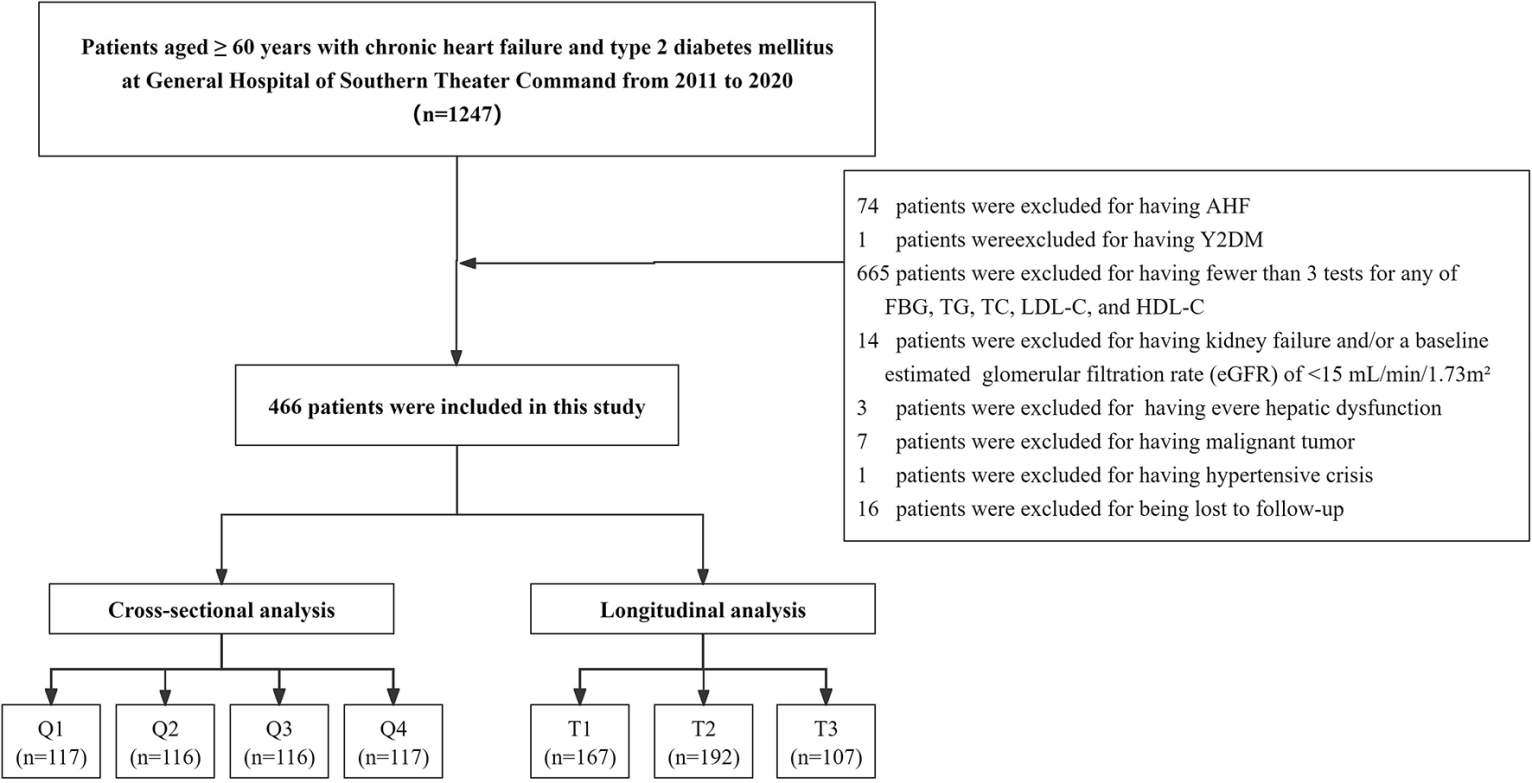
Flowchart of the study

In this study, 466 patients who visited the hospital from 2011–2020 were retrospectively recruited from electronic medical records at the General Hospital of the Southern Theater Command in Guangzhou, China. This study included 1,247 patients who met the following criteria: (1) aged ≥ 60 years; (2) had a clinical diagnosis of CHF [23]; and (3) had a clinical diagnosis of T2DM [24]. Of the 1,247 patients, 781 patients were excluded on the basis of the following criteria: (1) had acute heart failure (AHF) at baseline; (2) had type 1 diabetes (T1DM); (3) had fewer than 3 tests for fasting blood glucose (FBG), triglyceride (TG), total cholesterol (TC), low-density lipoprotein (LDL-C), and high-density lipoprotein (HDL-C) levels; and (4) had kidney failure and/or a baseline estimated glomerular filtration rate (eGFR) less than 15 mL/min per 1.73 square meters (m^2^); (5) had severe hepatic dysfunction at baseline; (6) had a malignant tumor at baseline; (7) had thyroid dysfunction (either hyperthyroidism or hypothyroidism) at baseline; and (8) had a hypertensive crisis at baseline. Finally, we established a cohort of 466 patients and grouped them according to both the baseline TyG index quartiles in the cross-sectional analysis and their TyG index trajectories in the longitudinal analysis (Fig. 1).

### Ethics statement

This research rigorously followed the guidelines of the Declaration of Helsinki and was approved by the Ethics Committee of the General Hospital of the Southern Theater Command (Project Ethics No. NZLLKZ2024135). Given the nature of this retrospective observational study, the Institutional Review Board waived the need for informed consent and guaranteed the confidentiality of all patient-identifying information.

### Data collection and definitions

We obtained the information from the electronic medical records system. This information was divided into disease history, medications, and laboratory tests. Body mass index (BMI) was calculated as weight divided by the square of height (kg/m^2^). Smoking was defined as having smoked or currently smoking, whether in the past or at present. Drinking was the same as smoking. The formula for calculating the TyG index was ln (fasting triglycerides [mg/dL] × fasting blood glucose [mg/dL]/2) [25].

The arrhythmia status includes atrial fibrillation, conduction block, sick sinus node, sinus bradycardia/tachycardia, ventricular tachycardia, ventricular flutter, ventricular fibrillation, et. In addition to common comorbidities, the disease history includes etiology of HF (classified as non-cardiac and cardiogenic) and duration of type 2 diabetes (DDM, time interval from diagnosis to enrollment). The categorized heart failure etiology was based on medical records. Patients diagnosed with ischemic heart disease, myocardial infarction, hypertension, valvular heart disease, arrhythmias, diabetes, or hyperthyroidism were classified as having cardiogenic heart failure. Conversely, those diagnosed with pulmonary disease, kidney disease, or other non-cardiac factors were deemed to have non-cardiogenic heart failure. The drug information includes the use of drugs for the treatment of HF, hypoglycemic drugs and lipid-lowering drugs at the time of enrollment, and the use of novel treatment drugs during follow-up (novel drugs for the treatment of HF(NTHF) was sacubitril valsartan sodium tablets, and new hypoglycemic drugs was dipeptidyl peptidase-4 and sodium-glucose cotransporter 2, SGLT2). In addition, we also collected information on the use of lipid-lowering medications. The laboratory information includes the NYHA classification, ejection fractions (EF), systolic blood pressure (SBP), diastolic blood pressure (DBP), BMI, total bilirubin (TBIL), direct bilirubin (DBIL), total protein (TP), albumin (ALB), albumin/globulin (A/G), alanine aminotransferase (ALT), alkaline phosphatase (ALP), total bile acid (TBA), adenosine deaminase (ADA), blood urea nitrogen (BUN), serum creatinine (Scr), uric acid (UA), TG, TC, LDLC, HDLC, apolipoprotein A_1_ (Apo-A_1_), apolipoprotein B (Apo-B), lipoproteins (LP[a]), glycosylated hemoglobin (HbA1C), FBG, brain natriuretic peptide (BNP), potassium (K), sodium (Na), chlorine (Cl), white blood cell (WBC), neutrophil (NEUT), lymphocyte (L), eosinophil (E), basophil (B), red blood cell (RBC), hemoglobin (HGB), hematocrit (HCT), mean corpuscular volume (MCV), mean corpuscular hemoglobin content (MCH), mean corpuscular hemoglobin concentration (MCHC), blood platelet (PLT), mean platelet volume (MPV), platelet distribution width (PDW), and platelet hematocrit (PCT).

### Follow-up and outcomes

The study ended in June 2024, and the average follow-up period for participants was 7.33 (range, 1.33–12.42) years. The main endpoint event of this study was WHF, as defined according to the 2023 National Heart Failure Guideline (Simplified Version) [2]. Specifically, WHF in patients with CHF must meet three criteria: (1) they must have had a period of clinical stability and been receiving consistent anti-heart failure treatment; (2) they must experience WHF symptoms and/or signs; and (3) they must require hospitalization for urgent evaluation and receive intravenous medication or other specialized treatment, with a definitive diagnosis confirmed by the clinical team. The secondary endpoint event of this study is a critical measure: overall death, which refers to any death regardless of its cause.

### Statistical analysis

In the cross-sectional analysis, patients were characterized according to baseline TyG index quartiles. In the longitudinal study, patients were subsequently grouped and described on the basis of the TyG index trajectory. Four distinct TyG index patterns were discerned via a linear mixed model (LMM): low decreasing TyG index trajectory (T1, n = 167, 35.8%), sustained medium TyG index trajectory (T2, n = 192, 41.2%), and high decreasing TyG index trajectory (T3, n = 107, 23.0%).

Continuous variables are reported as median and interquartile range (IQR) values. Differences between groups were analyzed via the Kruskal‒Wallis H test. Categorical variables are presented as frequencies and percentages (%). Differences between groups were analyzed via either the chi-square test or Fisher's exact test.

Competing risk Cox regression was used to assess the association between baseline TyG index and WHF and overall mortality. The competing risk Cox regression was used to assess the link between the TyG index trajectory and the hazards of WHF as well as overall mortality. Standard cox regression was used to assess the link between the TyG index and the hazards of overall mortality, and performs as one of the sensitivity analysis methods. These models were adjusted for multiple variables to account for potential confounding factors. Model 1 involved an analysis without any adjustments. In Model 2, adjustments were made for gender, age, smoking status, drinking status and BMI. Model 3 was built on the basis of gender, age, smoking status, drinking status, BMI, hypertension, arrhythmia, CHD, cerebral infarction, hyperuricemia, DDM, NTHF, hypoglycemic drugs, lipid-lowering drugs, NYHA classification, HbA1c, BNP, and EF. The variables entering the multivariate regression model first need to be preliminarily screened by the univariate competing risk Cox regression model, the lasso regression model, and the random forest model. The TyG index trajectory was incorporated only as a categorical variable. In all three models, the first quartile (Q1) of the TyG index and low decreasing TyG index trajectory (T1) were used as the reference group. In addition, restrictive cubic spline (RCS) regression was used to assess the nonlinear relationship between the baseline TyG index and the hazards of WHF and overall mortality. We used standard cox regression as the sensitivity analysis. Stratification analyses were performed basing on the standard Cox regression analysis.

In this study, we used R software (version 4.4.2, developed by the R Foundation for Statistical Computing in Vienna, Austria), Python software (version 3.13, provided by Python Software Foundation (PSF) in Amsterdam, Netherlands), and Stata software (version 18.0, provided by Computer Resource Center in Chicago, United States) for data analysis and statistical processing. A *P* value less than 0.05, which was determined via a two-tailed test, was deemed statistically significant.

## Results

### Cross-sectional feature analysis on the basis of baseline TyG index quartiles

Among the 466 patients included in this study, the median age was 74 (67, 81) years, and 186 patients (39.91%) were female. Table 1 displays the baseline characteristics stratified by the baseline TyG index quartiles. Compared with the Q1 group, the top quartile (Q4) group was found to have a greater proportion of females, younger individuals, a higher BMI, higher rate of hypoglycemic drugs, and increased comorbidity rates for hypertension, CKD, and CHD. However, this group had decreased comorbidity rates for arrhythmia. Notable disparities in several clinical indicators were observed among the groups. Higher levels of SBP, DBP, BMI, TP, ALP, ADA, BUN, TG, TC, LDL-C, Apo-A1, Apo-B, HbA1c, FBG, WBC, NEUT, L, E, RBC, MCHC, PLT, MPV, PDW, and PCT were observed in the Q4 group. Lower levels of TBIL, DBIL, ALB, A/G, HDLC, Na, Cl, MCV, and MCH were observed in the Q4 group.

**Table 1:** Cross-sectional feature analysis based on baseline TyG index quartiles

Variables	Total	Q1 (7.3–8.4)	Q2 (8.4–8.9)	Q3 (8.9–9.4)	Q4 (9.4–10.9)	*P* value
Number of patients	446	117	116	116	117	
Female, n (%)	186 (39.9)	36 (30.8)	36 (31.0)	54 (46.6)	60 (51.3)	< 0.001
Smoking, n (%)	100 (21.5)	30 (25.6)	24 (20.7)	23 (19.8)	23 (19.7)	0.645
Drinking, n (%)	41 (8.8)	13 (11.1)	9 (7.7)	8 (6.9)	11 (9.4)	0.680
Hypertension, n (%)	394 (84.6)	95 (81.2)	97 (83.6)	99 (85.3)	103 (88.0)	0.527
Arrhythmia, n (%)	223 (47.9)	72 (61.5)	65 (56.0)	45 (38.8)	41 (35.0)	< 0.001
CKD, n (%)	209 (44.9)	44 (37.6)	50 (43.1)	50 (43.1)	64 (54.7)	0.061
CHD, n (%)	388 (83.3)	92 (78.6)	103 (88.8)	96 (82.8)	97 (82.9)	0.224
Cerebral infarction, n (%)	164 (35.2)	38 (32.5)	45 (38.8)	35 (30.2)	46 (39.3)	0.363
Hyperuricemia, n (%)	140 (30.0)	27 (23.1)	36 (31.0)	38 (32.8)	39 (33.3)	0.288
Hyperlipidemia, n (%)	156 (33.5)	29 (24.8)	29 (25.0)	47 (40.5)	51 (43.6)	0.001
NYHA > II, n (%)	154 (33.0)	32 (27.4)	51 (44.0)	30 (25.9)	41 (35.0)	0.012
Etiology of HF, n (%)	431 (92.5)	109 (93.2)	110 (95.8)	107 (92)	105 (89.7)	0.518
Anti-heart failure drugs, n (%)	352 (75.5)	94 (80.3)	89 (76.7)	84 (72.4)	85 (72.6)	0.442
NTHF, n (%)	122 (26.2)	29 (20.5)	41 (28.4)	23 (14.7)	29 (16.2)	0.040
Hypoglycemic drugs, n (%)	412 (88.4)	97 (82.9)	99 (85.3)	103 (88.8)	113 (96.6)	0.007
New hypoglycemic drugs, n (%)	187 (40.1)	49 (41.9)	46 (39.7)	43 (37.1)	49 (41.9)	0.859
Lipid-lowering drugs, n (%)	364 (78.1)	90 (76.9)	91 (78.4)	89 (76.7)	94 (801.3)	0.902
DDM, years, (median [IQR])	8.0 (3.0, 10.0)	8.0 (3.0,10.0)	7.0 (3.0,10.0)	8.0 (3.0,10.0)	8.0 (2.0,10.0)	0.832
Age, years, (median [IQR])	74.0 (67.0, 81.0)	77.0 (69.0, 82.0)	76.0 (67.0, 83.0)	72.5 (67.0, 80.0)	71.0 (66.0, 76.0)	< 0.001
EF, %, (median [IQR])	60.0 (57.3, 67.0)	61.0 (59.0, 67.0)	60.5 (56.3, 67.0)	61.5 (60.0, 66.0)	60.0 (57.0, 67.0)	0.886
SBP, mmhg, (median [IQR])	135.0 (123.0, 150.0)	130.0 (120.0, 147.0)	133.0 (120.0, 146.0)	136.0 (123.5, 147.0)	135.0 (128.0, 154.0)	0.121
DBP, mmhg, (median [IQR])	74.0 (66.0, 81.8)	74.0 (64.5, 82.0)	71.0 (66.3, 80.0)	74.0 (65.0, 82.0)	75.0 (66.0, 82.0)	0.665
BMI, kg/m^2^, (median [IQR])	24.2 (22.4, 26.6)	24.2 (22.0, 26.4)	23.9 (22.5, 26.5)	23.9 (22.5, 26.3)	24.2 (22.3, 26.7)	0.641
TBIL, mol/L, (median [IQR])	10.8 (8.0, 14.8)	12.5 (9.5,16.4)	10.8 (8.3,15.3)	10.8 (7.9,13.3)	8.9 (6.9,12.7)	< 0.001
DBIL, mol/L, (median [IQR])	3.4 (2.4, 4.8)	3.8 (2.7, 5.6)	3.7 (2.7, 5.2)	3.1 (2.1, 4.4)	2.9 (1.9, 4.0)	< 0.001
TP, g/L, (median [IQR])	65.8 (61.8, 70.2)	64.2 (60.3, 68.3)	66.5 (63.0, 70.5)	65.7 (62.3, 70.2)	66.7 61.3, 70.6)	0.028
ALB, g/L, (median [IQR])	39.6 (36.7, 42.3)	39.1(37.0, 42.9)	39.8 (37.7, 42.0)	39.9 (36.7, 42.7)	39.0 (35.7, 41.9)	0.235
A/G, (median [IQR])	1.5 (1.3, 1.7)	1.6 (1.4, 1.8)	1.5(1.4, 1.7)	1.6 (1.3, 1.8)	1.5 (1.2, 1.7)	0.010
ALT, U/L, (median [IQR])	17.0 (12.0, 24.8)	15.0 (12.0, 22.0)	18.0 (12.0, 27.5)	17.0 (12.8, 24.0)	17.0 (12.0, 26.0)	0.231
ALP, U/L, (median [IQR])	62.0 (49.0, 77.0)	58.0 (48.0, 73.5)	64.0 (50.2, 76.5)	60.0(48.0, 72.8)	67.0(51.0, 80.0)	0.429
TBA, umol/L, (median [IQR])	3.8 (2.2, 6.5)	4.2 (3.0, 7.2)	3.7 (2.1, 6.8)	3.2(2.1, 6.4)	3.9 (2.2, 6.3)	0.345
ADA, U/L, (median [IQR])	13.0 (10.0, 17.0)	12.0 (9.0, 16.0)	14.0 (11.0, 17.0)	12.0 (9.0, 17.0)	14.0 (11.0, 19.0)	0.839
BUN, mmol/L, (median [IQR])	6.1 (4.9, 7.9)	5.7 (4.6, 7.2)	6.6 (5.1, 8.1)	5.7(4.8, 7.4)	6.4(5.1, 9.1)	0.013
Scr, umol/L, (median [IQR])	86.5 (70.0, 109.0)	84.0 (71.00,100.0)	93.0 (75.8, 117.0)	80.0 (65.0, 106.3)	87.0 (68.0, 116.0)	0.019
UA, umol/L, (median [IQR])	388.5 (320.0, 466.0)	369.0 (306.5, 437.0)	403.5 (329.8, 486.8)	395.0 (321.8, 456.8)	379.0 (305.0, 466.0)	0.136
TG, mmol/L, (median [IQR])	1.3 (1.0, 2.0)	1.3 (1.0, 1.9)	1.4 (0.9, 2.0)	1.3 (1.0, 1.9)	1.3 (1.0, 2.0)	< 0.001
TC, mmol/L, (median [IQR])	4.1 (3.4, 5.0)	3.6 (3.1, 4.4)	3.6 (3.2, 4.4)	4.3 (3.7, 5.4)	4.7 (3.8, 5.7)	< 0.001
LDLC, mmol/L, (median [IQR])	2.3 (1.7, 3.1)	1.95 (1.5, 2.5)	2.1 (1.6, 2.7)	2.55 (2.0, 3.6)	2.8 (2.1, 3.4)	< 0.001
HDLC, mmol/L, (median [IQR])	1.1 (0.9, 1.3)	1.2 (1.0, 1.4)	1.1 (0.9, 1.2)	1.2 (1.0, 1.3)	1.1 (0.9, 1.2)	< 0.001
Apo-A_1_, g/L, (median [IQR])	1.1 (1.0, 1.3)	1.1 (1.0, 1.3)	1.1 (0.9, 1.3)	1.2 (1.0, 1.3)	1.1 (1.0, 1.3)	0.142
Apo-B, g/L, (median [IQR])	0.8 (0.6, 1.0)	0.7 (0.5, 0.8)	0.7 (0.6, 0.9)	0.9 (0.7, 1.1)	1.0 (0.8, 1.2)	< 0.001
LP_(a)_, g/L, (median [IQR])	0.2 (0.1, 0.4)	0.2 (0.1, 0.4)	0.2 (0.1, 0.3)	0.2 (0.1, 0.4)	0.2 (0.1, 0.4)	0.527
HbA1c, %, (median [IQR])	6.7 (6.0–8.1)	6.0 (5.8–7.0)	6.3 (5.9–7.3)	6.8 (6.0–7.8)	7.9 (6.6–9.5)	< 0.001
FBG, mmol/L, (median [IQR])	6.4 (5.4, 8.1)	6.4 (5.3, 7.9)	6.4 (5.2, 7.9)	6.1 (5.3, 8.1)	6.4 (5.5, 8.6)	< 0.001
BNP, pg/mL, (median [IQR])	66.6 (32.2, 178.7)	69.7 (28.4, 144.0)	68.2 (35.7, 219.0)	63.0 (28.8, 165.0)	68.5 (33.0, 174.0	0.438
K, mmol/L, (median [IQR])	4.0 (3.7, 4.3)	4.0 (3.7, 4.2)	4.0 (3.7, 4.3)	4.0 (3.7, 4.3)	4.0 (3.8, 4.4)	0.588
Na, mmol/L, (median [IQR])	141.0 (139.0,143.0)	141.0 (140.0,143.0)	141.0 (139.0, 143.0)	141.0 (139.0, 143.0)	140.0 (137.0, 142.0)	0.001
Cl, mmol/L, (median [IQR])	103.0 (100.0,106.0)	104.0 (101.0,107.0)	103.0 (101.0, 107.0)	103.0 (100.0, 105.0)	102.0 (97.0, 104.0)	< 0.001
WBC, 10*9/L, (median [IQR])	7.3 (5.9, 9.0)	6.5 (5.0, 8.1)	7.3 (5.8, 9.0)	7.5 (6.3, 9.0)	8.1 (6.6, 9.8)	< 0.001
NEUT, 10*9/L, (median [IQR])	4.6 (3.5, 6.1)	3.9 (3.0, 5.2)	4.5 (3.4, 6.0)	4.6 (3.6, 6.2)	5.0 (4.2, 6.7)	< 0.001
L, 10*9/L, (median [IQR])	1.7 (1.3, 2.2)	1.6 (1.2, 1.9)	1.7 (1.4, 2.0)	1.8 (1.4, 2.3)	1.8 (1.4, 2.3)	0.004
Mono, 10*9/L, (median [IQR])	0.5 (0.4, 0.7)	0.5 (0.4, 0.6)	0.5 (0.4, 0.7)	0.5 (0.4, 0.7)	0.5 (0.4, 0.7)	0.096
E, 10*9/L, (median [IQR])	0.2 (0.1, 0.3)	0.1 (0.1, 0.2)	0.2 (0.1, 0.3)	0.2 (0.1, 0.2)	0.2 (0.1, 0.3)	0.571
B, 10*9/L, (median [IQR])	0.02 (0.01, 0.03)	0.02 (0.01, 0.03)	0.03 (0.02, 0.04)	0.02 (0.01, 0.03)	0.02 (0.02, 0.04)	0.009
RBC, 10*9/L, (median [IQR])	4.1 (3.7, 4.5)	4.1 (3.7, 4.5)	4.1 (3.6, 4.5)	4.1 (3.8, 4.6)	4.2 (3.7, 4.6)	0.152
HGB, g/L, (median [IQR])	123.0 (111.2, 137.0)	123.0 (113.0, 135.5)	123.0 (110.3, 134.8)	123.5 (110.0, 137.0)	123.0 (111.0, 140.0)	0.963
HCT, L/L, (median [IQR])	0.4 (0.3, 0.4)	0.4 (0.3, 0.4)	0.4 (0.3, 0.4)	0.4 (0.3, 0.4)	0.4 (0.3, 0.4)	0.912
MCV, fl, (median [IQR])	91.3 (87.7, 94.7)	92.6 (89.7, 96.3)	91.8 (88.6, 95.3)	91.0 (87.0, 94.1)	89.9 (86.0, 92.3)	< 0.001
MCH, pg, (median [IQR])	30.3 (29.1, 31.5)	30.7 (29.6, 31.9)	30.4 (29.4, 31.8)	30.1 (28.6, 31.2)	30.0 (28.9, 31.1)	0.004
MCHC, g/L, (median [IQR])	330.0 (322.0, 339.0)	329.0 (322.0, 337.5)	330.0 (321.0, 338.0)	328.0 (321.8, 336.0)	334.0 (325.0, 341.0)	0.019
PLT, 10*9/L, (median [IQR])	200.0 (162.2, 242.0)	183.0 (149.0, 218.5)	198.0 (160.3, 225.8)	201.0 (164.8, 260.3)	220.0 (177.0, 265.0)	< 0.001
MPV, 10*9/L, (median [IQR])	10.7 (10.1, 11.3)	10.6 (10.0, 11.2)	10.6 (10.0, 11.0)	10.6 (10.1, 11.2)	10.9 (10.2, 11.6)	0.065
PDW, 10*9/L, (median [IQR])	12.2 (10.9, 13.5)	12.1 (10.8, 13.2)	12.1 (10.9, 13.1)	12.2 (10.7, 13.6)	12.7 (11.3, 14.0)	0.080
PCT, 10*9/L, (median [IQR])	0.2 (0.2, 0.3)	0.2 (0.2, 0.2)	0.2 (0.2, 0.2)	0.2 (0.2, 0.3)	0.2 (0.2, 0.3)	< 0.001
WHF, n (%)	127 (27.3)	29 (24.8)	31 (26.7)	26 (22.4)	41 (35.0)	0.149
Overall mortality, n (%)	183 (39.3)	43 (36.8)	46 (39.7)	39 (33.6)	55 (47.0)	0.186

The 5-year incidence rates of WHF in Q1–Q4 were 13.7%, 8.6%, 9.5%, and 20.5%, respectively. The 10-year incidence rates of WHF in Q1–Q4 were 20.5%, 25.0%, 22.4%, and 35.0%, respectively. The incidence rates of WHF more than 10 years in Q1–Q4 were 24.8%, 26.7%, 22.4%, and 35.0%, respectively. The 5-year overall mortality rates in Q1–Q4 were 15.4%, 21.6%, 13.8%, and 26.5%, respectively. The 10-year overall mortality rates in Q1–Q4 were 29.9%, 37.1%, 32.8%, and 44.4%, respectively. The overall mortality rates more than 10 years in Q1–Q4 were 36.8%, 39.7%, 33.6%, and 47.0%, respectively. In terms of the baseline TyG index quartiles, significant differences were observed in the 5-year rates of WHF among the groups (*P* = 0.028).

### Risk of WHF based on the baseline TyG index

**Fig. 2:**
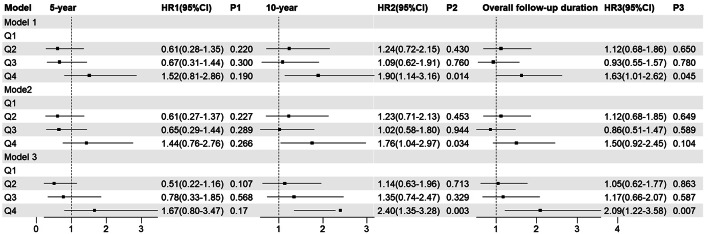
The risk of WHF based on the TyG index quartile. Model 1: unadjusted; Model 2: adjusted for gender, age smoking, drinking, and BMI; Model 3: adjusted for gender, age, smoking, drinking, BMI, hypertension, arrhythmia, CHD, cerebral infarction, hyperuricemia, DDM, NTHF, hypoglycemic drugs, lipid-lowering drugs, NYHA classification, HbA1c, BNP, and EF

Figure 2 displays the outcomes of the competing risk Cox regression (Fig. 2).

At 5-year follow-up, we observed no difference between groups in the TyG index quartile and the risk of WHF. At 10-year follow-up, the patient in Q4 had an increased risk of WHF, with the HRs of 1.90 (95% CI 1.14–3.16) in Model 1, 1.76 (95% CI 1.04–2.97) in Model 2, and 2.40(95% CI 1.35–3.28) in Model 3. During the overall follow-up duration, the outcomes were diverse, and we observed a significant association between the Q4 group and WHF with the HRs of 1.63 (95% CI 1.01–2.62) in the Model 1, and the HRs of 2.09 (95% CI 1.22–3.58) in the Model 3.

**Fig. 3:**
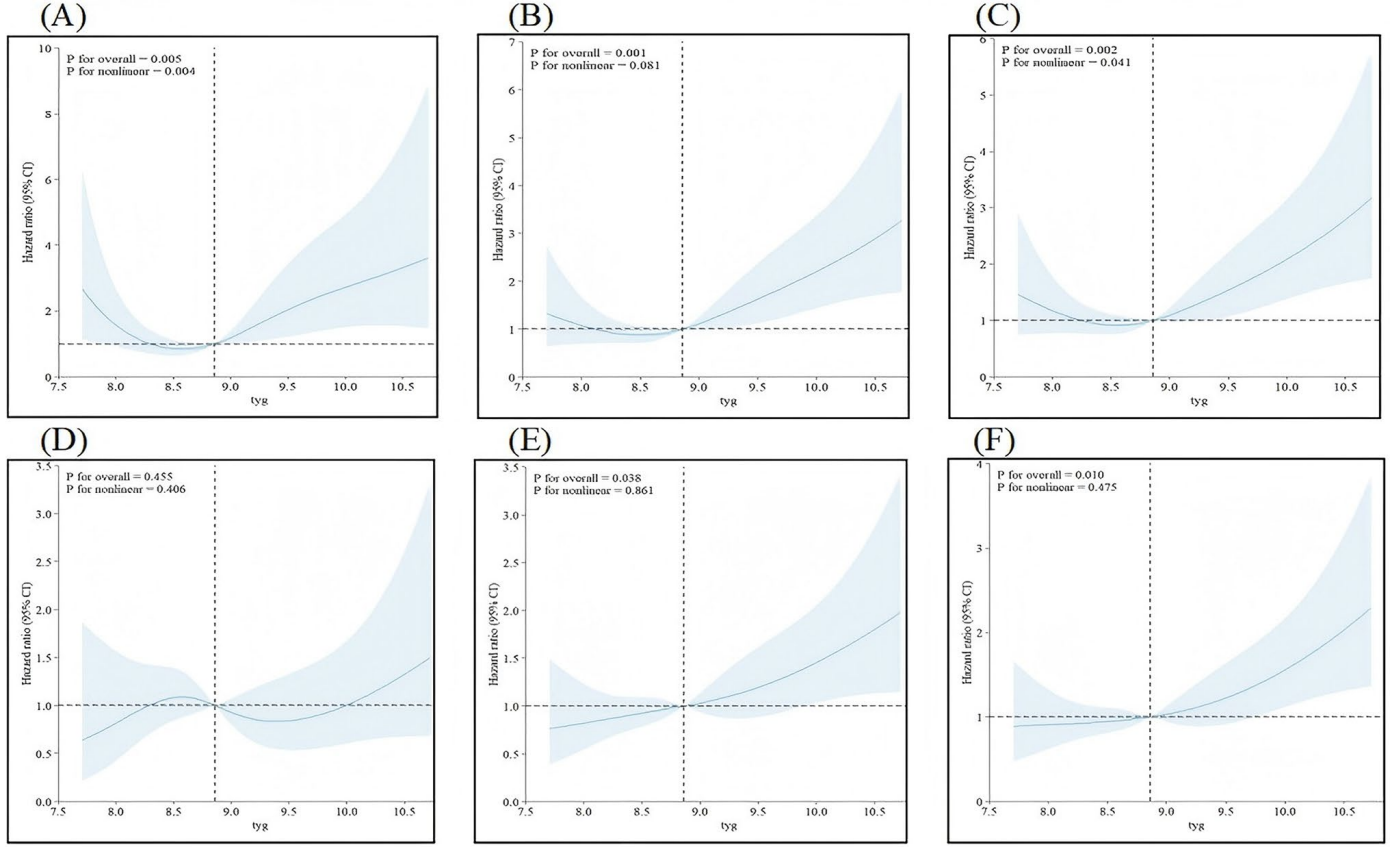
Dose‒response relationships between the TyG index and WHF as well as overall mortality. **A** For 5-year WHF; **B** For 10-year WHF; **C** For overall follow-up WHF; **D** For 5-year overall mortality; **E** For 10-year overall mortality; **F** For overall follow-up overall mortality

We used RCS regression on the basis of Model 3 to explore the dose‒response relationship between the TyG index and the hazards of WHF at different follow-up periods (Fig. 3A–C). A TyG index value of 8.86 was set as the baseline, with a corresponding HR of 1. This analysis demonstrated a J-shaped curve in the association of the TyG index with the risk of WHF (*P* < 0.05).

### Risk of overall mortality based on the baseline TyG index

**Fig. 4:**
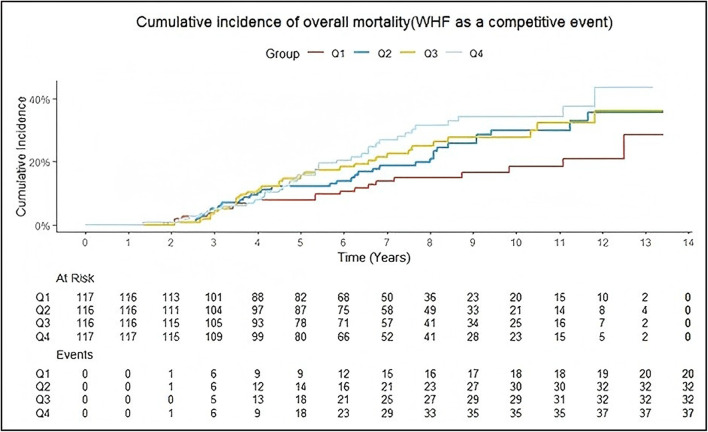
The CIF of overall mortality based on TyG index quartiles

**Fig. 5:**
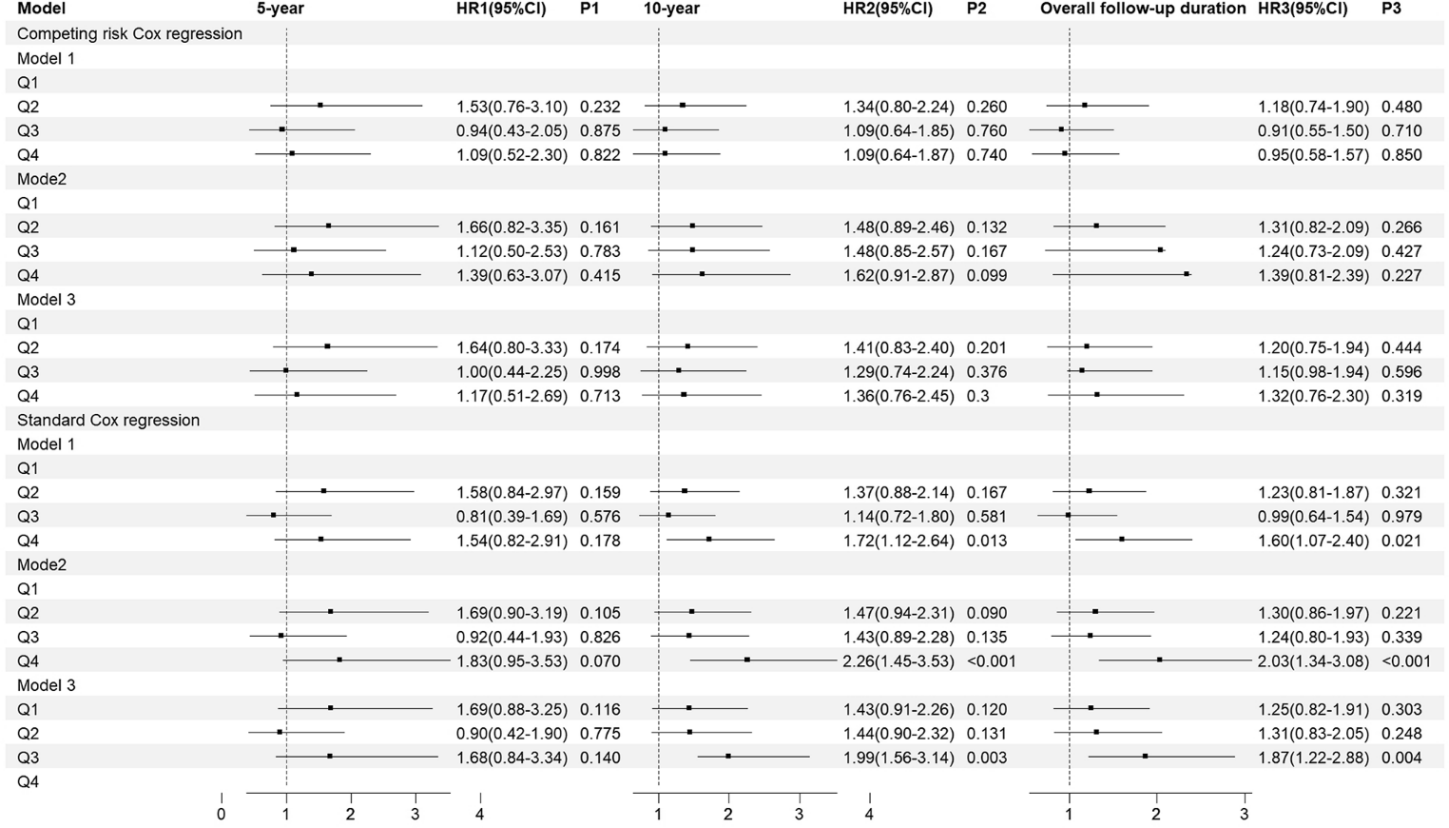
The risk of overall mortality based on TyG index quartiles. Model 1: unadjusted; Model 2: adjusted for gender,age smoking, drinking, and BMI; Model 3: adjusted for gender, age, smoking, drinking, BMI, hypertension, arrhythmia, CHD, cerebral infarction, hyperuricemia, DDM, NTHF, hypoglycemic drugs, lipid-lowering drugs, NYHA classification, HbA1c, BNP, and EF

The results of survival analysis with WHF as a competitive event were presented as cumulative incidence function (CIF) plot (Fig. 4). Similar to the baseline analysis, no statistically significant differences were observed in the comparison of overall mortality rates among the groups. In terms of cumulative overall mortality, at the 5-year follow-up, the Q1–Q4 group had a mortality rate of 7.7%, 12.0%, 16.0%, and 16.0% respectively(*P* = 0.300). At the 10-year follow-up, the Q1–Q4 group had a mortality rate of 19.0%, 30.0%, 28.0%, and 34.0% respectively (*P* = 0.090). Throughout the entire follow-up period, the Q1–Q4 group had a mortality rate of 29.0%, 36.0%, 36.0%, and 43.0% respectively (*P* = 0.067) (Table 2). In terms of the HRs and its 95%CI, we observed that there were no statistically significant differences in HRs between the groups (Fig. 5). When the competitive risk of WHF was not considered, there was a difference between the level of each TyG index and the risk of all-cause mortality over 10 years. And with or without adjustment for confounders, it was observed that the risk was higher in the group with a high baseline TyG index than in the group with a low level. At 10-year follow-up, the risk of overall mortality in the Q4 was 1.72 times (1.12–2.64) in model 1, 2.26 times (1.45–3.53) in model 2, and 1.99 times (1.56–3.14) in model 3, respectively. In terms of overall follow-up, the risk of overall mortality in the Q4 was 1.60 times (1.07–2.40) in model 1, 2.03-fold (1.34–3.08) in model 2, and 1.87-fold (1.22–2.88) in model 3 compared with the Q1 group, respectively.

**Table 2:** The cumulative incidence of overall mortality based on TyG index quartiles

Group	5-year		10-year		Overall follow-up duration	
	Overall mortality (%)	*P*	Overall mortality (%)	*P*	Overall mortality (%)	*P*
Q1	7.7	0.300	19.0	0.090	29.0	0.067
Q2	12.1		30.0		36.0	
Q3	15.5		28.0		36.0	
Q4	15.4		34.0		43.0

We used RCS regression on the basis of standard Cox regression Model 3 to explore the dose‒response relationship between the TyG index and the hazards of overall mortality at different follow- up periods (Fig. 3D–F). We demonstrated a linear relationship between the TyG index and the hazards of overall mortality (*P* > 0.05).

### Stratification and sensitivity analysis based on the TyG index quartile

To evaluate the influence of variables such as gender, age, and comorbidities on the link between the TyG index and study endpoints, stratification analyses were conducted. The results showed an interaction between BMI and TyG index when overall mortality was used as the endpoint event (Supplementary Material 1). The results revealed that male patients in Q4, under 80 years old, with hypertension, using NTHF and diabetes (including newer drugs), no drugs for the treatment of HF and BMI < 27 kg/m^2^, had a higher risk of WHF. In terms of all-cause mortality, the risk of overall mortality was higher in the Q4 compared with patients with Q1 in female under the age of 80, who have hypertension, CKD and hyperlipidemia and not used lipid-lowering drugs.

To see the stability of the model, we performed sensitivity analysis (Supplementary Material 2–3). Regarding the risk of WHF, the results of standard Cox regression were inconsistent only at the overall follow-up of Model 2. Specifically, standard Cox regression was observed at this time when the risk of heart failure worsening was higher in the Q4 than in Q1, with HR and 95% CI of 1.76 (1.07–2.89). We performed sensitivity analyses on multiple datasets, and the results showed that our model was robust in both the competitive risk Cox regression model and the standard Cox regression model.

### Baseline features based on longitudinal TyG index trajectories

**Fig. 6:**
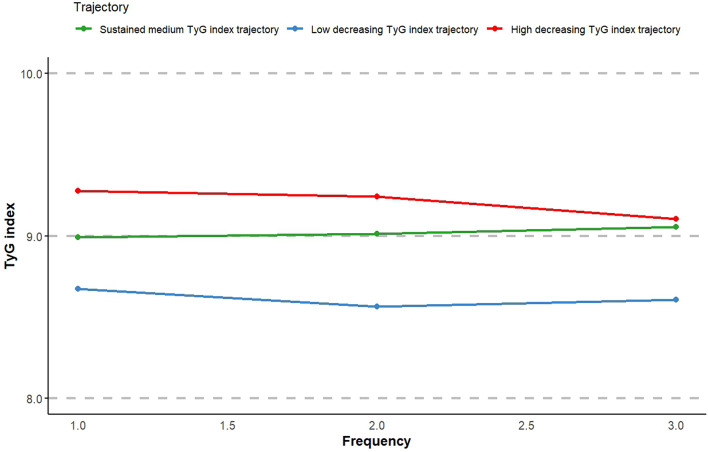
TyG index trajectory during follow-up

The 466 patients were categorized into three groups according to their TyG index trajectory: low decreasing TyG index trajectory (T1, n = 167, 35.8%), sustained medium TyG index trajectory (T2, n = 192, 41.2%), and high decreasing TyG index trajectory (T3, n = 107, 23.0%) (Fig. 6). The baseline features derived from the longitudinal trajectories of the TyG index are displayed in Table 3. The baseline TyG index had a median value of 8.3(8.1, 8.7) for the T1 group, 9.0(8.6, 9.3) for the T2 group, and 9.8 (9.4, 10.2) for the T3 group. Compared with the T1 group, the T3 group had a greater percentage of females and a lower median age. Additionally, the T3 group presented elevated levels of the following medical parameters: SBP, TP, ALP, ADA, BUN, TG, TC, LDL-C, Apo-B, HbA1c, WBC, NEUT, L, MCV, PLT, MPV, PDW, and PCT. During the follow-up duration, the rates of WHF and overall mortality were higher in the T3 group, with 33.6% and 48.6% respectively.

**Table 3:** Baseline features based on longitudinal TyG index trajectories

Variables	T1 (N = 167)	T2 (N = 192)	T3 (N = 107)	*P* value
TyG index, (median [IQR])	8.3 (8.1, 8.7)	9.0 (8.6, 9.3)	9.8 (9.4, 10.2)	< 0.001
Female, n (%)	45 (26.9)	86 (44.8)	55 (51.4)	< 0.001
Smoking, n (%)	37 (22.2)	39 (20.3)	20 (18.7)	0.781
Drinking, n (%)	11 (6.6)	18 (9.4)	5 (4.8)	0.295
Hypertension, n (%)	138 (82.6)	161 (83.9)	95 (88.8)	0.366
Arrhythmia, n (%)	101 (60.5)	84 (43.8)	38 (35.5)	< 0.001
CKD, n (%)	67 (40)	84 (43.8)	57 (53.3)	0.100
CHD, n (%)	139 (83.2)	155 (80.9)	94 (87.9)	0.287
Cerebral infarction, n (%)	58 (3437)	66 (34.4)	40 (37.4)	0.862
Hyperuricemia, n (%)	43 (29.7)	62 (32.3)	35 (32.7)	0.318
Hyperlipidemia, n (%)	42 (25.2)	70 (36.5)	50 (46.7)	< 0.001
NYHA > II, n (%), n (%)	51 (30.5)	70 (36.5)	33 (30.8)	0.424
Cause of HF	168 (93.9)	188 (92.2)	75 (90.4)	0.590
Anti-heart failure drugs, n (%)	137 (82.0)	136 (70.8)	79 (73.8)	0.044
NTHF, n (%)	44 (24.6)	35 (17.2)	14 (16.9)	0.143
Hypoglycemic drugs, n (%)	143 (85.6)	168 (87.5)	101 (96.4)	0.076
New hypoglycemic drugs, n (%)	72 (40.2)	78 (38.2)	37 (44.6)	0.610
Lipid-lowering drugs, n (%)	141 (78.8)	157 (76.9)	66 (79.5)	0.647
DDM, years, (median [IQR])	6.00 (2.00, 10.00)	5.00 (2.00, 9.00)	6.00 (1.50, 10.00)	0.138
Age, years, (median [IQR])	76.0 (68.0, 81.5)	74.0 (67.0, 81.0)	72.0 (66.5, 79.0)	0.010
EF, %, (median [IQR])	62.0 (56.5, 66.0)	62.0 (58.8, 68.0)	62.0 (57.0, 68.0)	0.249
SBP, mmhg, (median [IQR])	131.0 (120.0, 146.0)	135.0 (124.0, 150.0)	136.0 (128.0, 159.0)	0.053
DBP, mmhg, (median [IQR])	72.0 (65.5, 80.0)	75.0 (65.0, 82.0)	74.0 (66.0, 82.0)	0.870
BMI, kg/m^2^, (median [IQR])	23.9 (22.4, 26.2)	24.2 (22.4, 27.0)	24.4 (22.4, 26.7)	0.580
TBIL, mol/L, (median [IQR])	11.9 (8.9, 15.8)	10.7 (8.0, 14.7)	8.8 (6.9, 12.2)	< 0.001
DBIL, mol/L, (median [IQR])	3.7 (2.7, 5.6)	3.4 (2.3, 4.4)	2.9 (1.8, 3.9)	< 0.001
TP, g/L, (median [IQR])	64.6 (61.2, 69.0)	65.7 (62.2, 70.6)	67.1 (63.9, 70.1)	0.025
ALB, g/L, (median [IQR])	39.3 (37.0, 42.2)	39.8 (37.0, 42.2)	39.4 (35.8, 42.0)	0.293
A/G, (median [IQR])	1.6 (1.4, 1.8)	1.5 (1.3, 1.7)	1.5 (1.2, 1.7)	0.030
ALT, U/L, (median [IQR])	16.0 (12.0, 24.0)	17.5 (12.0, 27.0)	17.0 (12.5, 23.0)	0.215
ALP, U/L, (median [IQR])	60.0 (49.5, 72.5)	62.0 (49.0, 76.2)	67.0 (54.0, 77.5)	0.037
TBA, umol/L, (median [IQR])	3.80 (2.35, 6.35)	3.80 (2.20, 6.60)	3.30 (2.15, 5.30)	0.317
ADA, U/L, (median [IQR])	13.0 (10.0, 16.0)	12.0 (10.0, 17.0)	16.0 (12.0, 19.0)	0.155
BUN, mmol/L, (median [IQR])	6.0 (4.8, 7.5)	5.9 (4.6, 7.9)	6.6 (5.4, 8.7)	0.082
Scr, umol/L, (median [IQR])	88.0 (74.0,106.0)	84.0 (68.0, 107.0)	88.0 (68.0, 122.0)	0.627
UA, umol/L, (median [IQR])	388.0 (310.0, 476.0)	391.0 (326.0, 466.0)	382.0 (305.0, 454.0)	0.285
TG, mmol/L, (median [IQR])	1.3 (0.9, 2.0)	1.3 (1.0, 2.0)	1.4 (1.0, 2.0)	0.739
TC, mmol/L, (median [IQR])	3.6 (3.1, 4.4)	4.2 (3.5, 5.3)	4.6 (3.7, 5.5)	< 0.001
LDLC, mmol/L, (median [IQR])	2.0 (1.5, 2.8)	2.5 (1.9, 3.2)	2.6 (2.0, 3.3)	< 0.001
HDLC, mmol/L, (median [IQR])	1.1 (1.0, 1.3)	1.1 (1.0, 1.3)	1.1 (0.9, 1.2)	0.060
Apo-A_1_, g/L, (median [IQR])	1.1 (1.0, 1.3)	1.1 (1.0, 1.3)	1.1 (0.9, 1.2)	0.046
Apo-B, g/L, (median [IQR])	0.7 (0.5, 0.8)	0.8 (0.7, 1.0)	0.9 (0.8, 1.2)	< 0.001
LP_(a)_, g/L, (median [IQR])	0.2 (0.1, 0.3)	0.2 (0.1, 0.3)	0.2 (0.1, 0.3)	0.855
HbA1c, %, (median [IQR])	6.1 (5.9–7.0)	6.9 (6.0–7.9)	8.3 (6.6–9.8)	< 0.001
FBG, mmol/L, (median [IQR])	6.5 (5.3, 7.9)	6.1 (5.3, 8.2)	6.5 (5.5, 8.8)	0.245
BNP, pg/mL, (median [IQR])	69.4 (35.7, 168.0)	65.4 (31.4, 179.0)	66.4 (31.8, 179.0)	0.843
K, mmol/L, (median [IQR])	4.0 (3.7, 4.3)	4.0 (3.7, 4.3)	4.1 (3.8, 4.4)	0.064
Na, mmol/L, (median [IQR])	141.0 (139.0, 143.0)	141.0 (139.0, 143.0)	139.0 (137.0, 142.0)	< 0.001
Cl, mmol/L, (median [IQR])	103.0 (102.0, 106.0)	103.0 (99.0, 106.0)	102.0 (98.0, 103.0)	< 0.001
WBC, 10^9^/L, (median [IQR])	6.7 (5.3, 85)	7.4 (6.1, 9.3)	7.8 (6.7, 9.7)	< 0.001
NEUT, 10^9^/L, (median [IQR])	4.3 (3.2, 5.6)	4.6 (3.6, 6.5)	5.2 (4.2, 6.6)	< 0.001
L, 10^9^/L, (median [IQR])	1.6 (1.3, 1.9)	1.7 (1.4, 2.3)	1.8 (1.3, 2.4)	< 0.001
Mono, 10^9^/L, (median [IQR])	0.5 (0.4, 0.6)	0.5 (0.4, 0.7)	0.5 (0.4, 0.7)	0.054
E, 10^9^/L, (median [IQR])	0.11 (0.07, 0.22)	0.15 (0.09, 025)	0.18 (0.09, 0.31)	0.229
B, 10^9^/L, (median [IQR])	0.02 (0.01, 0.03)	0.02 (0.01, 0.03)	0.02 (0.02, 0.04)	0.174
RBC, 10^9^/L, (median [IQR])	4.1 (3.7, 4.5)	4.1 (3.7, 4.5)	4.3 (3.8, 4.7)	0.182
HGB, g/L, (median [IQR])	124.0 (112.0, 136.0)	123.0 (112.0, 136.0)	125.0 (109.0, 140.0)	0.738
HCT, L/L, (median [IQR])	0.38 (0.34, 0.41)	0.38 (0.34, 0.41)	0.38 (0.34, 0.42)	0.924
MCV, fl, (median [IQR])	92.1 (88.5, 95.6)	91.4 (88.3, 94.3)	88.7 (85.2, 91.4)	< 0.001
MCH, pg, (median [IQR])	30.5 (29.5, 31.8)	30.2 (29.2, 31.4)	29.8 (28.4, 31.2)	< 0.001
MCHC, g/L, (median [IQR])	329.0 (322.0, 338.0)	330.0 (322.0, 338.0)	333.0 (322.0, 340.0)	0.553
PLT, 109/L, (median [IQR])	190.0 (154.0, 222.0)	200.0 (164.0, 251.0)	219.0 (172.0, 266.0)	< 0.001
MPV, 109/L, (median [IQR])	10.5 (10.0, 11.1)	10.7 (10.2, 11.3)	10.9 (10.2, 11.6)	0.011
PDW, 109/L, (median [IQR])	11.9 (10.6, 13.0)	12.2 (11.1, 13.5)	12.8 (11.3, 14.3)	< 0.001
PCT, 109/L, (median [IQR])	0.20 (0.17, 0.23)	0.21 (0.18, 0.26)	0.23 (0.20, 0.29)	< 0.001
WHF, n (%)	41 (24.6)	50 (26.0)	36 (33.6)	0.227
Overall mortality, n (%)	56 (33.5)	75 (39.1)	52 (48.6)	0.045

### Risk of WHF based on TyG index trajectories

**Fig. 7:**
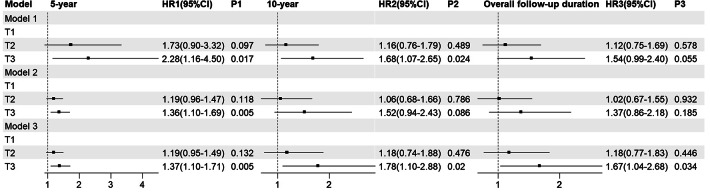
The risk of WHF based on TyG index trajectories. Model 1: unadjusted; Model 2: adjusted for gender,age smoking, drinking, and BMI; Model 3: adjusted for gender, age, smoking, drinking, BMI, hypertension, arrhythmia, CHD, cerebral infarction, hyperuricemia, DDM, NTHF, hypoglycemic drugs, lipid-lowering drugs, NYHA classification, HbA1c, BNP, and EF

Figure 7 demonstrated the risk of WHF based on TyG index trajectories (Fig. 7). The results of the competing risk Cox regression analysis show that the association between different TyG index trajectories and the occurrence of WHF was observed in 5-year and 10-year follow-up period. At 5-year follow-up, we found that the risk of WHF in T3 was 2.28 times (1.16–4.50) in model 1, 1.36-fold (1.10–1.69) in model 2, and 1.37-fold (1.10–1.71) in model 3, respectively, compared with T1. At 10-year follow-up, a difference in the risk of WHF was observed only in model 1 and model 3, with HR and 95% CI of 1.68 (1.07–2.65) in model 1 and 1.78 (1.10–2.88) model 3, respectively. At overall ollow-up, a higher risk was observed in the T3 than in the T1 only in the fully adjusted model 3.

### Risk of overall mortality based on TyG index trajectories

**Fig. 8:**
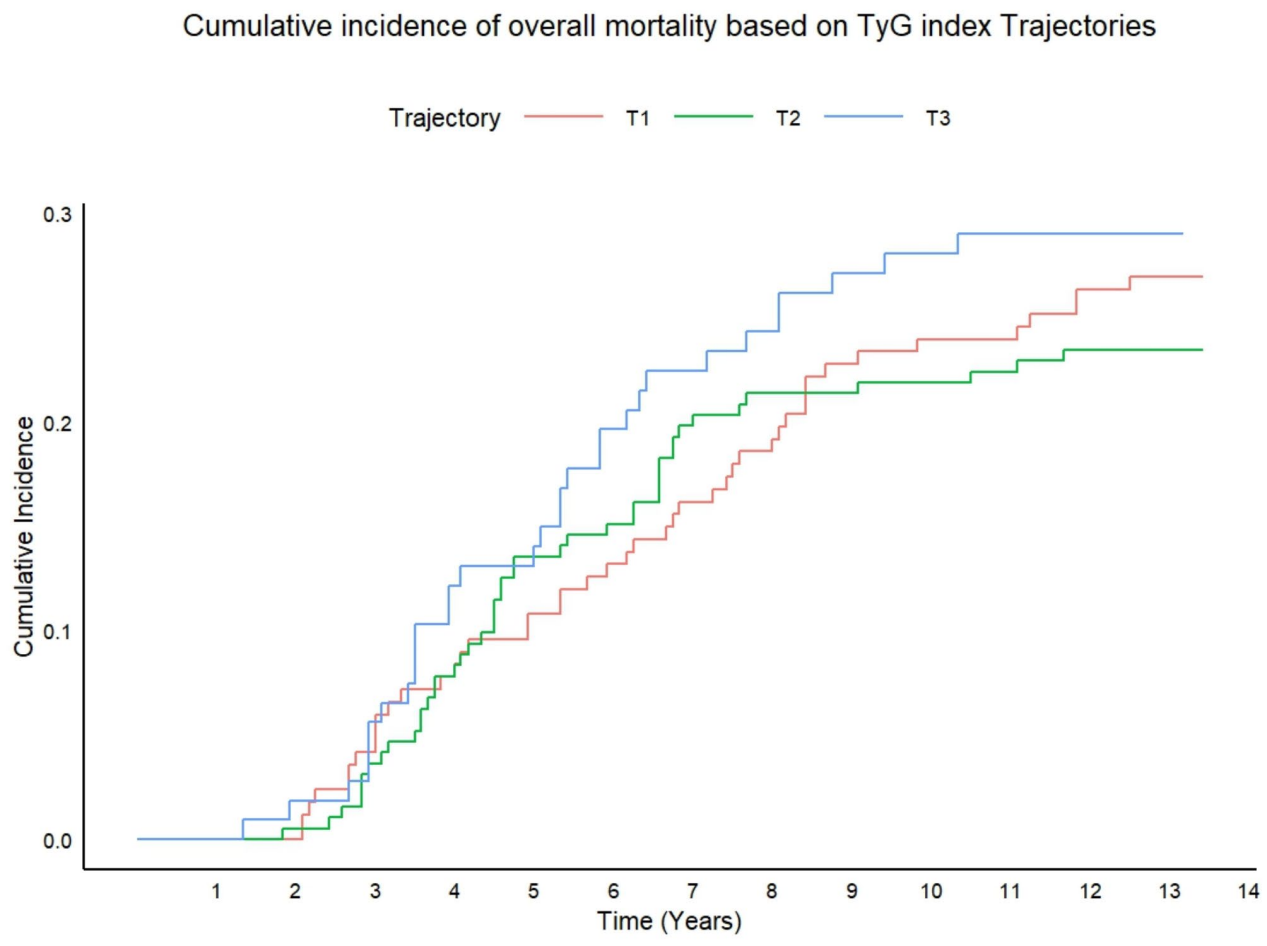
The CIF of overall mortality based on TyG index trajectories

**Fig. 9:**
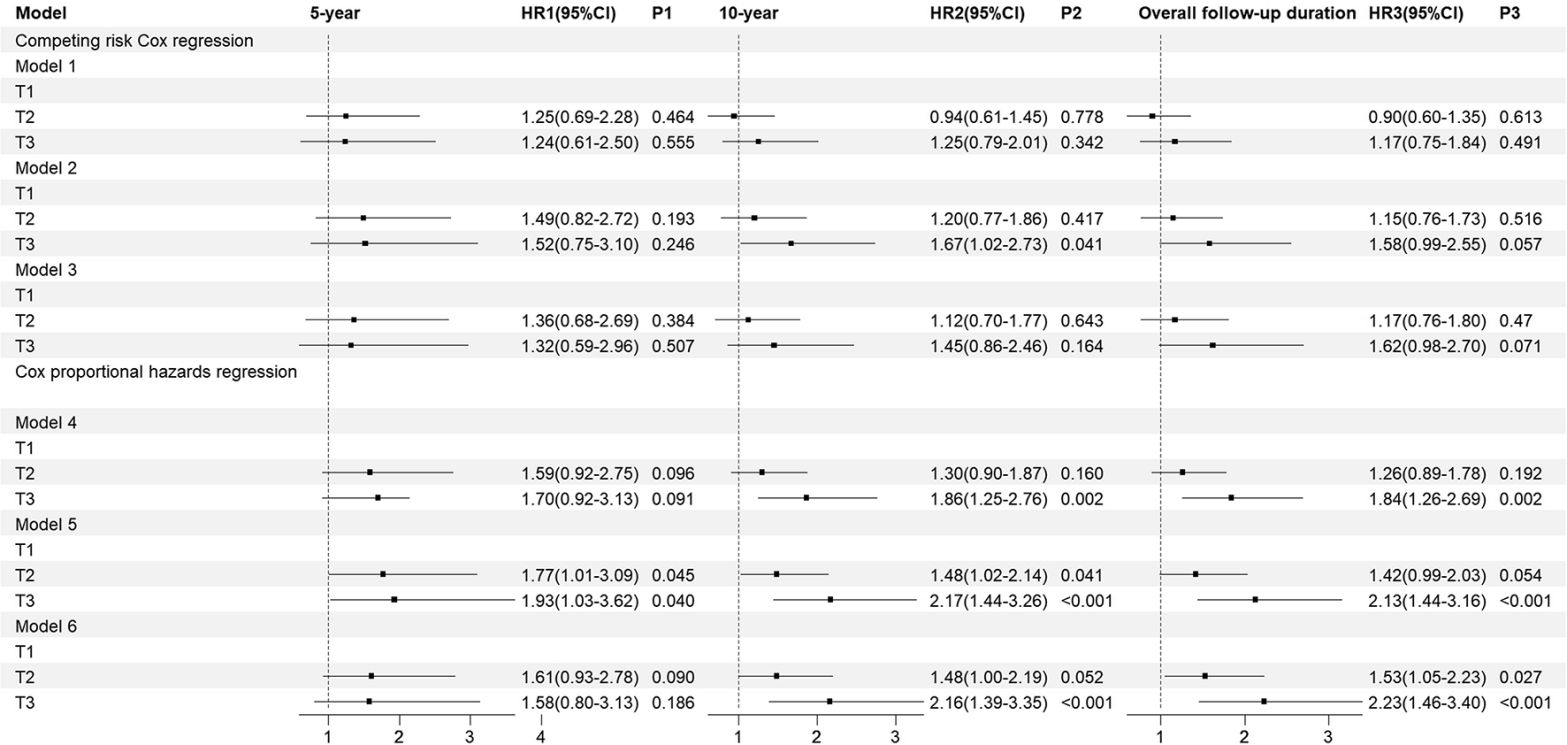
The risk of overall mortality based on TyG index trajectories. Model 1 and Model 4: unadjusted; Model 2 and Model 5: adjusted for gender, age smoking, drinking, and BMI; Model 3 and Model 6: adjusted for gender, age, smoking, drinking, BMI, hypertension, arrhythmia, CHD, cerebral infarction, hyperuricemia, NYHA classification, drugs for the treatment of DM, NTHF, Statins, DDM, HbA1c, BNP, and EF

Figure 8 displayed the CIF plot for overall mortality with WHF as the competing event. Table 4 presented the cumulative overall mortality rates for different follow-up durations in this model. Neither the CIF plot nor the competing risks Cox regression analysis revealed significant differences among the TyG index trajectories (Fig. 9). However, However, when the competitive risk of WHF was not considered, a statistically significant difference in the risk of overall mortality over 10 years was observed across the TyG index trajectories. The 10-year risk of overall mortality in the T3 was 1.86-fold (1.25–2.76) in model 1, 2.17-fold (1.44–3.26) in model 2, and 2.16-fold (1.39–3.35) in model 3, higher than in T1, respectively. This phenomenon was also observed at overall follow-up.

**Table 4:** The cumulative incidence of overall mortality based on TyG index trajectories

Group	5-year	10-year	Overall follow-up duration
	Overall mortality (%)	Overall mortality (%)	Overall mortality (%)
T1	10.1	24.0	26.9
T2	13.5	21.9	23.4
T3	13.1	28.000	29.0

### Stratification and sensitivity analysis based on TyG index trajectories

The results of the stratification analysis showed that an interaction between NTHF and TyG index trajectory when overall mortality was used as the endpoint event (Supplementary Material 1). In terms of the risk of WHF, the patients in the T3 group, using of NTHF and no using of drugs for the treatment of HF and NTDM, NYHA > 2, BMI < 27 was observed to had a higher risk of WHF than in the T1 group. In terms of the risk of overall mortality, females under 80 years of age in the T3, with hypertension, CKD, using hypoglycemic drugs and NTHF was observed to had a higher risk of overall mortality than in the T1 group. In addition, the risk of overall mortality in the T3 group was higher than that in the T1 group in the hyperlipidemia, rugs for the treatment of HF, NTDM, and lipid-lowering drugs stratification (Supplementary Material 4).

In terms of sensitivity analysis, multiple datasets were validated, which showed that the competing risk regression model was robust (Supplementary Material 5–6).

## Discussion

This cohort study, with a median follow-up of 7.33 years, presents the following key findings in the TyG index and its trajectory and elderly patients with CHF and T2DM: (1) When considering the competitive risk of overall mortality, patients with high baseline TyG index was associated with an increased risk of WHF over a 10-year or longer period. When the competitive risk of WHF was not considered, a high baseline TyG index increases the risk of overall mortality for more than 10 years. (2) A J-shaped nonlinear pattern was noted linking the baseline TyG index to the risk of WHF, whereas a linear correlation was found with overall mortality during the 10-year or longer follow-up period. (3) The high decreasing trajectory of the TyG index was associated with an increased risk of WHF within a decade. And, when the competitive risk of WHF was not considered, a high decreasing trajectory of the TyG index increases the risk of overall mortality for more than 10 years.

### Correlation of the TyG index and its trajectory with WHF

IR is a critical characteristic of disorders of glucose and lipid metabolism and is recognized as a significant predictor of CVD development and progression. The TyG index is a useful indicator of IR and is commonly employed to evaluate the prognosis of patients with CVD and other conditions.

Research has indicated that the TyG index is linked to the risk of HF [26, 27]. A 31-year multicenter study revealed that the TyG index during adolescence served as an independent predictor of congestive HF onset, and for every extra unit of the TyG index, there was a 2.8-fold greater likelihood of developing congestive HF [28]. Guo's research indicated that, among individuals suffering from CHF and T2DM who were in the intensive care unit, the TyG index was associated with a heightened risk of cardiovascular mortality and HF-related rehospitalization[29]. Fu discovered that the TyG index was linked to HF hospitalizations in DM patients older than 65 years who received right ventricular pacing (RVP) and additionally reported a J-shaped nonlinear correlation between the TyG index and the likelihood of HF hospitalizations [30]. We found that the TyG index was positively correlated with prolonged WHF in elderly patients with CHF and T2DM. And during the 5-year follow-up interval, the relationship between the TyG index quartile and the risk of WHF was rarely observed. A major reason for this is that the WHF was caused by the long-term accumulation of CHF and comorbidities. Outcome in terms of baseline TyG index was similar to the conclusions of Zhou et al., indicating that the TyG index is associated with long-term prognostic indicators such as all-cause mortality, cardiovascular mortality, and HF readmission in patients with HFpEF [39]. Furthermore, we revealed a “J”-shaped nonlinear correlation of the TyG index with WHF, thereby echoing the findings of the study conducted by Fu [30]. Gender is a crucial factor in the prognosis of HF, with females being at greater risk of HF-related negative outcomes due to their increased likelihood of having HF risk factors [31].Wang reported that a high TyG index elevated the likelihood of CVDs in elderly individuals by approximately 20–30% compared with a low baseline TyG index [32]. In our study, males aged ≤ 80 years had a greater risk of WHF. This is in partial contrast to earlier studies, and there may be two reasons. First, the included patients were not grouped according to reduced ejection fraction, retention, or borderline ejection fraction, and women were more likely to have heart failure with preserved ejection fraction [33]. Second, a significant proportion of male patients who have previously worked in special occupations may have experienced worse living conditions, stress, depression, etc., which makes them more prone to adverse events [34].

In addition, we found that a high decreasing TyG index correlated with WHF over a 10-year period. Multiple studies Many studies have found similar result. A study from the National Health and Nutrition Examination Survey found that insulin resistance was associated with a 10-year Risk of Hard Cardiovascular Event over a decade [35]. And a high-level TyG index is an effective tool for time-based assessment of insulin resistance. In hypertension cohort studies, a rapidly elevated TyG index in adolescents was correlated with a greater likelihood of arteriosclerosis, with an odds ratio (OR) of 2.76 (95% CI 1.40–7.54) [21]. A study from Kailuan successfully predicted the occurrence of CVDs in elderly individuals via five TyG index trajectories [36]. After lifestyle factors were considered, an elevated TyG index trajectory was observed to increase the likelihood of CVDs by 2.72 times [37]. Zheng et al. demonstrated that participants with consistently high TyG levels were more prone to developing HF over a 10-year period than were those in the low-stable group [38]. A 12-year longitudinal study revealed that women with a “moderately increased” or “highly stable” TyG index trajectory were considerably more prone to hypertension than men were [39]. A study of 10,367 individuals revealed that a moderately stable TyG trajectory was independently associated with coronary artery stenosis (CAS) in participants, whereas a high-growth trajectory showed no notable correlation [40]. A study from Fujian revealed that, in comparison with the low-stable trajectory, the likelihood of frailty among individuals aged 60 years and above rose by 2.17 times (95% CI 1.01–3.88) in the high-stable trajectory [41]. A 10-year longitudinal study revealed that hypertensive individuals with an increasing TyG index tended to have increased susceptibility to ischemic stroke [22]. In our study, when considering the competitive risk of overall mortality, a high decreasing TyG index trajectory showed increase in the risk of WHF. Therefore, regular tracking of the changes in the TyG index in elderly patients with CHF and T2DM could aid in identifying individuals at increased risk of WHF.

### TyG index and its trajectory in relation to the risk of overall mortality

Studies have shown that in patients with HF, higher TyG index values are associated with a 1.5–2.3-fold increased risk of overall mortality within five years, in contrast to individuals with the lowest TyG index values [16, 42–44]. A Korean study revealed that male HF patients aged 70 years and above had a 1.30-fold greater risk of out-of-hospital death than women with HF did [45]. A Japanese study reported that among patients with AHF, the overall risk of mortality for females was 0.84 times greater than that for males [46]. Yuan et al.'s subgroup study revealed that female HF patients had a 1.31-fold greater mortality risk than male patients did [47]. A 478-day cohort study of 932 patients with AHF revealed a significant link between a higher TyG index in women and the risk of overall mortality [43]. A retrospective study of patients with AHF revealed a nonlinear relationship between the TyG index and overall death [46]. Among the general population, a J-shaped nonlinear pattern was observed in the dose‒response association between the TyG index and overall mortality risk [48]. However, we presented a linear relationship between the TyG index and long-term overall mortality.

Our findings revealed that in the absence of a competitive event, a high decreasing TyG index trajectory was positively correlated with overall mortality. The study by Shi et al. revealed that individuals with higher TyG index trajectories experienced at least a 1.2-fold elevation in the risk of major coronary events in comparison to those with a low trajectory, following adjustment for common risk factors [49].

Although the connection between the TyG index and overall mortality is complex, there are several possible explanations for this relationship. First, metabolic disturbances in triglycerides due to consistently high TyG index levels induce the accumulation of oxidative stress, ultimately leading to endothelial dysfunction. This disease is closely linked to adverse cardiovascular outcomes in HF patients [50]. Second, persistent hyperglycemia triggers oxidative stress, which activates protein kinase C and upregulates angiotensin II (Ang II) signaling within the heart. This cascade of events leads to vascular dysfunction, myocardial hypertrophy, fibrosis, and proinflammatory responses, highlighting the potential detrimental effects of chronic high blood sugar on the cardiovascular system [51]. Furthermore, the gradual increase in IR triggered the release of increased amounts of insulin and growth hormone-like peptides, such as insulin-like growth factor-1. This stimulates cell growth and suppresses cell death, thus increasing the likelihood of cancer development [52].

Compared with previous studies, this study has distinguishing features. We have proved that the TyG index trajectories were related to the rates of WHF as well as overall mortality in elderly patients with CHF and T2DM. By analyzing the dynamic changes in IR over time, this study provides a reference for improving the limitations of relying only on single-point measurements. Furthermore, this study presents an in-depth analysis of the differences in WHF and overall mortality risks in elderly patients with CHF and T2DM, considering factors such as age, gender, and comorbidities. This approach provided a more comprehensive understanding of the impact of IR on the risk of adverse outcomes in patients over 60 years of age with CHF and T2DM.

We also note the limitations present.

First, this study was limited to a single center, necessitating an expansion of the sample size and the inclusion of multiple research centers for external validation to ensure the generalizability of the findings. Second, as a retrospective cohort study, it did not comprehensively account for other time-varying factors beyond the TyG index, including smoking habits, alcohol consumption, and socioeconomic status. Owing to the minimal variability in these lifestyle factors and the inherent complexity of modeling them accurately, only baseline values were utilized in the analysis. Finally, our study was limited by population limitations, preventing the inclusion of a larger sample size. To increase the robustness of causal relationships, future research should focus on conducting large-scale prospective cohort studies.

## Conclusion

For elderly patients with CHF and T2DM, a high baseline TyG index levels increased the risk of WHF. And a high decreasing TyG index levels during follow-up were linked to an elevated risk of WHF within a decade and risk of overall mortality for more than 10 years. These findings highlight that continuous triglyceride-glucose index monitoring is crucial for reducing WHF and mortality risks in elderly patients with CHF and T2DM.

## Supplementary Information

Supplementary Material 1

Supplementary Material 2

Supplementary Material 3

Supplementary Material 4

Supplementary Material 5

Supplementary Material 6

## Data Availability

No datasets were generated or analysed during the current study.

## Abbreviations

CVDs: Cardiovascular diseases
HF: Heart failure
CHF: Chronic heart failure
AHF: Acute heart failure
WHF: Worsening of heart failure
T2DM: Type 2 diabetes
T1DM: Type 1 diabetes
II: Insulin insensitivity
IR: Insulin resistance
TyG: Triglyceride-glucose
LMM: Linear Mixed Model
CKD: Chronic kidney disease
CHD: Coronary heart disease
IQR: Interquartile range
KM: Kaplan‒Meier
HR: Hazard ratio
CI: Confidence interval
BNP: Brain natriuretic peptide
FBG: Fasting blood glucose
NYHA Classification: New York Heart Association Classification
DDM: Duration of type 2 diabetes
NTHF: Novel drugs for the treatment of HF
NTDM: Novel drugs for the treatment of DM
SGLT2: Sodium-glucose cotransporter 2
CIF: Cumulative incidence function
SBP: Systolic blood pressure
DBP: Diastolic blood pressure
BMI: Body mass index
TBIL: Total bilirubin
DBIL: Direct bilirubin
TP: Total protein
ALB: Albumin
A/G: Albumin/Globulin
ALT: Alanine aminotransferase
ALP: Alkaline phosphatase
TBA: Total bile acid
ADA: Adenosine deaminase
BUN: Blood urea nitrogen
Scr: Serum creatinine
UA: Uric acid
TG: Triglycerides
TC: Total cholesterol
LDLC: Low-density lipoprotein
HDLC: High-density lipoprotein
Apo-A_1_: Apolipoprotein A_1_
Apo-B: Apolipoprotein B
LP(a): Lipoproteins
HbA1C: Glycosylated hemoglobin
K: Potassium
Na: Sodium
Cl: Chlorine
WBC: White blood cell
NEUT: Neutrophils
L: Lymphocyte
E: Eosinophil
B: Basophil
RBC: Red blood cell
HGB: Hemoglobin
HCT: Hematocrit
MCV: Mean corpuscular volume
MCH: Mean corpuscular hemoglobin content
MCHC: Mean corpuscular hemoglobin concentration
PLT: Blood platelet
MPV: Mean platelet volume
PDW: Platelet distribution width
PCT: Platelet hematocrit
Q1: First quartile
Q4: Top quartile
T1: Consistently low-level trajectory
T2: Consistently intermediate-level trajectory
T3: Consistently high-level trajectory
AIC: Akaike information criterion
BIC: Bayesian information criterion
